# A consensus-based checklist for the critical appraisal of cost-of-illness (COI) studies

**DOI:** 10.1017/S0266462323000193

**Published:** 2023-06-16

**Authors:** Lena Schnitzler, Tracy E. Roberts, Louise J. Jackson, Aggie T. G. Paulus, Silvia M. A. A. Evers

**Affiliations:** 1 Department of Health Services Research, Care and Public Health Research Institute (CAPHRI), Faculty of Health, Medicine and Life Sciences (FHML), Maastricht University, Maastricht, The Netherlands; 2 Health Economics Unit, Institute of Applied Health Research, College of Medical and Dental Sciences, University of Birmingham, Birmingham, UK; 3 School of Health Professions Education (SHE), Faculty of Health, Medicine and Life Sciences (FHML), Maastricht University, Maastricht, The Netherlands; 4 Trimbos Institute, Netherlands Institute of Mental Health and Addiction, Utrecht, The Netherlands

**Keywords:** Checklist, cost-of-illness, economic burden, burden of disease, critical appraisal

## Abstract

**Objectives:**

To develop a consensus-based checklist that can be used as a minimum standard to appraise the comprehensiveness, transparency and consistency of cost-of-illness (COI) studies. This is important when, for instance, reviewing and assessing COI studies as part of a systematic review or when building an economic model.

**Methods:**

The development process of the consensus-based checklist involved six steps: (i) a scoping review, (ii) an assessment and comparison of the different checklists and their questions, (iii) the development of a (preliminary) checklist, (iv) expert interviews, (v) the finalization of the checklist, and (vi) the development of guidance statements explaining each question.

**Results:**

The result was a consensus-based checklist for the critical appraisal of COI studies, comprising seventeen main questions (and some additional subquestions) across three domains: (i) study characteristics; (ii) methodology and cost analysis; and (iii) results and reporting. Guidance statements were developed describing the purpose and meaning behind each question and listing examples of best practice. The following answer categories were suggested to be applied when answering the questions in the checklist: *Yes, Partially, No, Not Applicable*, or *Unclear.*

**Conclusions:**

The consensus-based checklist for COI studies is a first step toward standardizing the critical appraisal of COI studies and is one that could be considered a minimum standard. The checklist can help to improve comprehensiveness, transparency and consistency in COI studies, to address heterogeneity, and to enable better comparability of methodological approaches across international studies.

## Background

Cost-of-illness (COI) studies can help identify, measure, and value the economic burden an illness or disease can impose on society (1). It is a useful decision-making tool as their estimates can be used as a foundation for projecting disease expenses and a framework to address a certain health problem, among others (2;3). COI studies are a commonly used tool to provide researchers and policy/decision makers with relevant information regarding the different cost components and cost categories (or sectors) associated with an illness or disease and can describe healthcare spending as well as costs beyond healthcare (e.g., intersectoral costs) (3).

In order to allow for COI studies to optimally inform researchers and policy/decision makers, these studies need to be methodologically sound (4;5). Various checklists and guidance tools exist for full economic evaluations including, for instance, the Drummond Methods for the Economic Evaluation of Health Care Programmes (6), the Consensus on Health Economic Criteria checklist (CHEC-list) (7), and others. These checklists and guidelines play an important role in assessing the (methodological or reporting) quality of economic evaluations and are widely used. In comparison, there is an evident lack of guidance for COI studies and poor consensus on how to review and assess those studies and what tool(s) to use for critical appraisal (2;8–12).

To the best of our knowledge, there are only two tools that are specifically designed to assist in developing and assessing COI studies (13;14). Both tools require a deeper level of technical and methodological detail and are extensive in length. The issue of length is critical because a checklist is often expected to be rigorous but also practical to use. The objective of one of the two tools, the Checklist for the Development and Assessment of Cost-of-Illness Studies by Mueller et al., was to develop a checklist in German and specifically for the German context (14). The objective of the second tool, a Guide to Critical Evaluation by Larg and Moss, was to develop a guide for understanding and evaluating COI studies (13). However, it is unclear whether this guide was developed based on consensus and expert opinion.

Methodological approaches for COI studies can differ in a variety of aspects (e.g., objectives, study perspective, costs included, time horizon), giving rise to considerable methodological heterogeneity (15;16). This makes comparability across COI studies difficult and the assessment of the generalizability or transferability of study results almost impossible. Because of the lack of available tools to review and assess existing COI studies, researchers often develop their own one-off list of questions as part of their work (e.g., literature reviews).

An internationally applicable, standardized checklist is needed in English to review and critically appraise the methodological approaches taken and reported in a COI study, to assess a study’s comprehensiveness, transparency and consistency, to reflect on a study’s strengths and weaknesses, and to potentially increase comparability across COI studies.

### Aims and Objectives

The aim of this paper was to develop a consensus-based checklist that can be used as a minimum standard to appraise the comprehensiveness, transparency, and consistency of COI studies. This is important when, for instance, reviewing and assessing COI studies for example as part of a systematic review or when building an economic model.

## Methods

**Figure 1.**
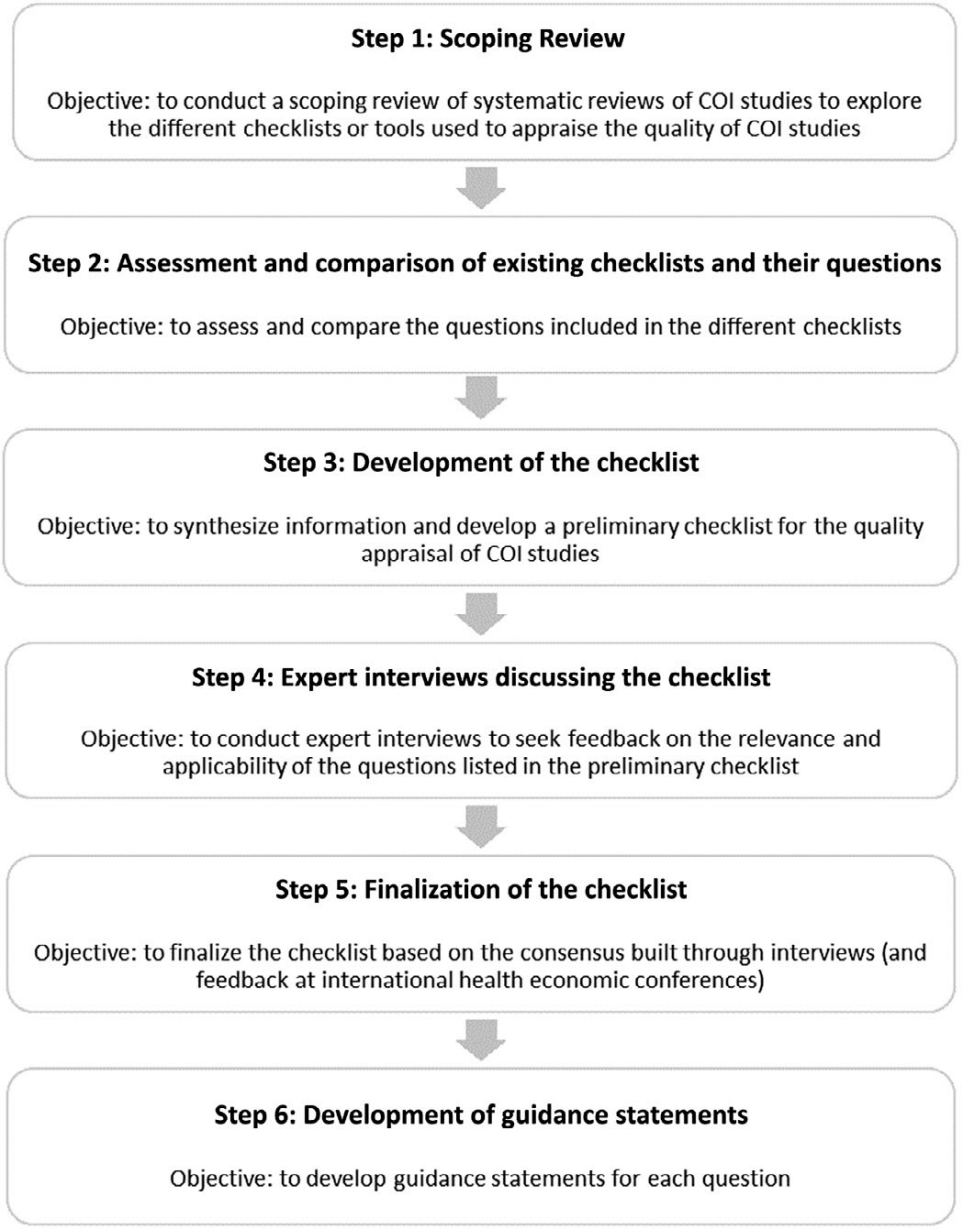
Development process of the consensus-based checklist.

The development process of the consensus-based checklist involved six sequential steps, as presented in Figure 1. These steps were based on previous approaches to the development of other relevant checklists and guidelines in health economics and related areas (7;14;17).

### Scoping Review

A targeted scoping review of systematic reviews of COI studies was conducted in MEDLINE (Ovid) to explore the different checklists or other tools used. This was complemented by hand searching and searches in Google Scholar, checking the reference lists of included articles, and reviewing articles, studies, checklists, and guidelines suggested by experts working in health economics and with COI studies. A search strategy was developed in MEDLINE using keywords and terminology relating to cost-of-illness, burden of illness, economic burden, systematic review(s), and checklist(s), focusing on papers published in 2010 (Supplementary Table 1). The search strategy combined search terms using Boolean operators “AND” and “OR” and searched for these keywords and terms in a paper’s title, abstract, and beyond. This date was selected as a previous study had considered papers prior to this date, which were reviewed (14). Studies were included in the scoping review that reported on applying at least one checklist or a similar tool for the quality or critical appraisal of COI studies. The aim was to identify all checklists and tools used by researchers for the quality or critical appraisal of COI studies, even if these checklists were not specifically designed for COI studies.

### Assessment and Comparison of the Different Checklists and Their Questions

The different checklists identified in the scoping review in Step 1 were listed, compared, and critically reviewed to determine whether they had been specifically designed for COI studies or were based on other existing health economic guidelines. This involved developing a matrix, charting all the questions and subquestions included in the identified checklists, to allow discussion and comparison by all authors. Due to the fact that not all checklists identified in Step 1 were specifically designed for COI studies (for example they may be for full economic evaluations), the questions (or criteria) included in these different checklists were carefully and critically reviewed in terms of their applicability and relevance for COI studies to identify and synthesize a set of key questions for assessing these studies. This meant that questions that were listed in existing checklists but were only applicable to full economic evaluations were excluded.

### Development of a Checklist for COI Studies

This step involved synthesizing the output of the scoping review (Step 1) and the results of the critical assessment (Step 2) to determine key areas that would need to be included in a checklist for COI studies. This was further refined to develop a list of the key questions that would be relevant and applicable and that could be used as a minimum standard for the critical appraisal of COI studies. From here on this will be referred to as the ‘preliminary checklist’. This provided an initial outline for discussion with the experts engaged in COI studies in the next step.

### Expert Interviews

Semi-structured, open-ended interviews were conducted with health economists and other experts from different countries working with COI studies to seek their expert opinion on the preliminary checklist and potentially identify questions to be added, removed, or revised. This process is fully described in a separate paper https://doi.org/10.1017/S0266462323000181. In this study, we use the term ‘experts’ to refer to individuals that are knowledgeable in a particular area, in this case in health economics/COI studies, and are/were actively involved in doing research around COI studies, including professors, assistant or associate professors, research fellows, among other. Experts were selected purposively based on their knowledge and expertise in relation to COI studies using network and snowball sampling. Interviews were audio-recorded, with the participant’s consent, and anonymized. A Framework approach was applied for the thematic analysis of the interviews, following systematic steps (18): interview recordings were transcribed verbatim by one author (L.S.); transcripts were entered and coded in NVivo, identifying themes and subthemes (L.S.); a set of transcripts and the coding framework were cross-checked by another author (L.J.); both authors familiarized themselves with the transcripts and agreed on a final framework listing relevant themes and sub-themes; the framework and findings were discussed among the author team (A.P., L.J., L.S., S.E., T.R.); findings were reported narratively. More detailed information on the methodology, conduct, and analysis of the interviews is provided in a separate paper https://doi.org/10.1017/S0266462323000181.

### Finalization of the Checklist

Experts’ feedback, suggestions, and recommendations on the preliminary checklist were carefully considered. The checklist was modified based on the experts’ feedback, removing certain questions, adding relevant questions, and rephrasing other questions, where applicable. The checklist was also presented at internal seminars in the Health Economics Unit at the University of Birmingham and at international health economics conferences, including the lolaHESG 2021 (The lowlands Health Economists’ Study Group) and the iHEA Conference 2021 (International Health Economics Association) to seek further feedback from experts in health economics. This step also involved the development of a list and description of answer categories suggested for use when answering the questions in the checklist, based on discussions with experts during the interviews and at the international conferences.

### Development of Guidance Statements

Guidance statements were developed for each question listed in the checklist to provide further information on the purpose and meaning behind each question and to give an example of a best practice. These guidance statements were based on existing health economic guidelines and best practices, to align the language and terminology in the checklist with the existing economic literature (3;4;7;19–22).

### Ethical Approval

Ethical approval to conduct this study was obtained from the University of Birmingham (ERN_20-1240).

## Results

The result was a consensus-based checklist for the critical appraisal of COI studies covering relevant questions in relation to study characteristics, methodology and cost analysis, and results and reporting (Table 1). Guidance statements explaining the questions and suggested answer categories were also established (Table 2). This study further generated relevant interview findings that are summarized and presented in a separate paper https://doi.org/10.1017/S0266462323000181.

**Table 1.** The consensus-based checklist for cost-of-illness studies

Item	Question	Answer^a^	Supportive information
Study characteristics			
Question/objective	Is a well-defined research question or objective stated?		
Population	Is the study population described?		
Perspective	a) Is (are) the chosen study perspective(s) stated?		
	b) If so, is (are) the chosen study perspective(s) justified?		
Methodology and cost analysis			
Epidemiological approach	Is the epidemiological approach reported (e.g., prevalence, incidence)?		
Costing approach	Is the costing approach reported (e.g., top-down, bottom-up)?		
Data collection approach	Is the data collection process reported (e.g., prospective, retrospective)?		
Identification	a) Are all components of resource use identified that are relevant to the condition/disease, population, intervention, study objectives, and study perspective?		
	b) If not, is a justification provided for excluding relevant components of resource use?		
Measurement	a) Are all included components of resource use measured?		
	b) If not, is a justification provided for not measuring certain components of resource use?		
Valuation	a) Are all included components of resource use valued in monetary terms?		
	b) If not, is a justification provided for not valuing certain components of resource use?		
Time horizon	a) Is the chosen time horizon specified?		
	b) If so, is the chosen time horizon justified?		
Discounting	a) Are future costs discounted?		
	b) If so, is a justification provided for the discount rate?		
Sensitivity	a) Are all variables whose values are uncertain subjected to sensitivity analysis?		
	b) If so, is a justification provided for which variables are subjected to sensitivity analysis?		
	c) Are analyses done on relevant subgroups?		
Results and reporting			
Cost sectors	Are the study results presented transparently by cost category/sector?		
Generalizability	Do the authors discuss the generalizability of study results (e.g., comparing the results to other patient/client groups or/in other settings)?		
Limitations	Do the authors discuss important limitations?		
Ethical and distributional issues	a) Do the authors discuss ethical issues?		
	b) Do the authors discuss distributional issues?		
Conflict of interest	Do the authors report any potential conflicts of interest?		

a Suggested answer categories: Yes, No, Partially, Not Applicable (NA), and Unclear. See Table 2 for further detail and guidance.

**Table 2.** Guidance statements

Item	Question	Guidance
Study characteristics		
1. Question/objective	Is a well-defined research question or objective stated?	A research question or objective should be stated and identify the study population and the type of disease(s)/condition(s) that is being assessed. The objective (or purpose) for why this study is conducted and needed should be described and be economically important (e.g., why this study is important to decision makers). The objective of the study ultimately determines the study perspective and subsequently resources captured in the analysis. Ideally, the research question or objective should include the chosen study perspective and indicate the costs that are being assessed.
2. Population	Is the study population described?	The study population should be described including information on patient characteristics (e.g., age, sex, ethnicity), geographic location, and clinical characteristics (e.g., disease stage, previous treatments, comorbidities). The study population described should be consistent with the population data in the study analysis. This information should be relevant to the motivation and objective of the study.
3. Perspective(s)	a) Is (are) the chosen study perspective(s) stated?	The study perspective(s) to address the research question or objective should be clearly stated. The study perspective ultimately depends on the study objective and stakeholder interests (e.g., government, provider, payer, decision/policy maker). The study needs to be specific about whether it assesses the economic burden of, for instance, society as a whole or a particular agent (e.g., provider, payer). The study perspective should ideally include a description of what payers are included. Examples: Societal, provider, health system, government, other
	b) If so, is (are) the chosen study perspective(s) justified?	A clear justification should be provided for the chosen study perspective as it determines the cost components to be included in the analysis. For example, the study might have followed national guidelines or reference cases. In some cases, national guidelines recommend adopting a narrower (e.g., healthcare, payer) perspective. The authors should always justify why a narrower perspective was applied and is valid.
Methodology and cost analysis		
4. Epidemiological approach	Is the epidemiological approach reported (e.g., prevalence, incidence)?	The epidemiological approach should be clearly reported. The prevalence approach estimates the economic burden of a disease/condition over a defined period of time (usually 1 year). The incidence approach estimates the economic burden of a disease/disease over (usually) over a lifetime.
5. Costing approach	Is the costing approach reported (e.g., top-down, bottom-up)?	The data quantification method should be clearly reported. The top-down approach uses aggregated data to estimate the attributable resources. The bottom-down approach uses individual-level data to estimate the quantity of inputs used and the unit costs of the inputs used.
6. Data collection	Is the data collection process reported (e.g., prospective, retrospective)?	The data collection process should be clearly reported. Cost-of-illness studies can be performed either prospectively or retrospectively depending on the relationship between the start of the study and the data collection. In a retrospective study, the events and resources have already occurred, and data has already been collected by the time the study is initiated. The previously collected data is then used for analysis. In a prospective study, the events and resources have not yet occurred by the time the study is initiated. This requires data to be collected (e.g., by following individuals) over time.
7. Identification of resource(s)	a) Are all components of resource use^a^ identified that are relevant to the condition/disease, population, intervention, study objectives, and study perspective?	A full identification and documentation of relevant resources should be provided. This includes the identification and inclusion of different categories of costs (e.g., healthcare, productivity). The definition of “relevant” costs is dependent on the disease/condition, study objective, and study perspective. Each cost component should be clearly stated. Where a study adopted more than one perspective (e.g., healthcare and societal perspective), the inclusion of the different resources under each perspective should be reported separately. Recommended subitems: Healthcare, individual/family, productivity losses, and other sectors (e.g., education, criminal justice)
	b) If not, is a justification provided for excluding relevant components of resource use?	A clear justification should be provided for excluded resources, including potentially relevant cost categories and cost components.
8. Measurement of resource(s)	a) Are all identified and included components of resource use measured?	Ideally, all identified and included resources should be measured. The methods (sources or instruments) for obtaining and quantifying the different components of resource use should be valid and clearly stated (e.g., interview, questionnaire, survey, cost-diary). If relevant, it should be stated if only costs specific to the disease/condition were included or if additional or excess costs were measured (e.g., costs related to comorbidities).
	b) If not, is a justification provided for not measuring certain components of resource use?	A justification should be provided for those resources that were not (or could not be) measured.
9. Valuation of resource(s)	a) Are all included components of resource use valued in monetary terms?	The sources of valuation for each unit price of every component of resource use should be valid and clearly stated. The currency and costing/reference year should be stated. The different approaches to valuing costs should be justified (e.g., what approach was taken to measure and value productivity costs, and why). If relevant, price adjustments over time should be reported. If relevant, it should be reported if prices were taken from other countries.
	b) If not, is a justification provided for not valuing certain components of resource use?	A justification should be provided for those resources that were not (or could not be) valued.
10. Time horizon	a) Is the chosen time horizon specified?	The chosen time horizon should be clearly stated. It refers to the period of analysis over which resources are assessed and is often associated with the choice of the epidemiological approach for analysis (e.g., prevalence, incidence). The time horizon should be long enough to capture all resources relevant to the disease/condition.
	b) If so, is the chosen time horizon justified?	A clear justification should be provided for the chosen time horizon. For example, the study might have followed national guidelines or reference cases.
11. Discounting	a) Are future costs discounted?	Where discounting is applicable the method for discounting future costs should be stated. Discounting indicates that costs that occur at different points in time (e.g., present costs and future costs) are valued differently. Hence, the timing of when costs incur plays a role when discounting future costs. For example, discounting is crucial for a study that adopted a time horizon longer than 1 year, that is, when applying an incidence approach where costs are estimated over a lifetime. Where studies used a time horizon of less than 1 year it might not be applicable to discount future costs.
	b) If so, is a justification provided for the discount rate?	The discount rate used in the study should be justified. For example, the study might have followed national guidelines or reference cases.
12. Sensitivity	a) Are all variables whose values are uncertain subjected to sensitivity analysis?	All variables in the analysis are potential candidates for the sensitivity analysis and should be presented or discussed. Different types of sensitivity analysis can include, for example, univariate and multivariate sensitivity analysis.
	b) If so, is a justification provided for which variables are subjected to sensitivity analysis?	A clear justification should be provided describing the range of the variables used in the sensitivity analysis. Only variables that are certain or which have a minimal impact on the study results (based on the preliminary analysis) can be excluded from the sensitivity analysis.
	c) Are analyses done on relevant subgroups?	Resource use or costs can vary across populations and subgroups (e.g., disease severity, gender, age, and ethnicity). In other words, characteristics of subgroups can influence the resource use or costs. Such heterogeneity of subgroups should be explored and, where relevant, separate analyses should be done on subgroups. For example, healthcare costs may be higher for males compared to females, older people compared to younger people, or other.
Results and reporting		
13. Cost sectors	Are the study results presented transparently by cost category/sector?	The presentation of cost components for each cost category is dependent on the study perspective. Where a study adopted more than one perspective (e.g., healthcare and societal perspective), the inclusion of the different cost components under each perspective should be reported separately. Recommended subitems: Healthcare, individual/family, productivity losses, other sectors (e.g., education, criminal justice)
14. Generalizability	Do the authors discuss the generalizability of study results (e.g., comparing the results to other patient/client groups or/in other settings)?	Generalizability refers to the applicability of the study results based on a (patient/client) sample to another sample (or setting). The study should clearly describe how research findings could be applied to other patient/client groups or settings and indicate how particular findings (or costs) could vary by patient/client groups, population, setting, location, care provider, or other.
15. Limitations	Do the authors discuss important limitations?	The study should discuss relevant limitations. Limitations can relate to, for instance, certain data, sources, cost components, assumptions, and (measurement, valuation) methods chosen for analysis. The reader should be able to understand the choice for certain methods and their main limitations. Recommended subitems: data, sources, cost components, assumptions, methods, other
16. Ethical and distributional issues	a) Do the authors discuss ethical issues?	Where applicable, the study should note ethical aspects that may raise some controversy. For example, placing a value on life/health and the methodological approach to do so may raise some ethical issues.
	b) Do the authors discuss distributional issues?	Where applicable, the study should elaborate on the characteristics of the population experiencing the disease/condition (young, old, poor, and wealthy) and how this may have distributional implications.
17. Conflict of interest	Do the authors report any potential conflict of interest?	The study should declare whether there is a potential conflict of interest. For example, a study should declare if an external agency financed the study to guarantee transparency in the relationship between the researcher and sponsor.
Answer categories		
Yes	Yes can be applied to indicate if a study reported on the requested information.	
No	No can be applied to indicate if a study missed to report on the requested information.	
Partially	Partially can be applied to indicate when a question was addressed or mentioned, but the information is not clearly or only suboptimally described.	
Not Applicable (NA)	Not Applicable (NA) can be applied to indicate where a question might not be applicable to a cost-of-illness study or in the context of the study.	
Unclear	Unclear can be applied when a question is addressed but it is not clear how exactly.	

a Other international literature might also refer to the components of resource use as “resources” or “costs.”

### Scoping Review

The scoping review of systematic reviews of COI studies published between 2010 and 2020 identified twenty-six studies that reported to have used a checklist or similar tool to assess COI studies. Six different checklists and guidelines were identified: the BMJ Checklist, the CHEC-list, the CHEERS checklist, the Drummond 10-point checklist, and the Drummond Methods for the Economic Evaluation of Health Care Programmes, and the Guide to Critical Evaluation by Larg and Moss (6;7;13;19;23). (The Drummond 10-point checklist is adapted from the Drummond Methods, but we followed the study authors’ ways of reporting). A seventh tool was identified through handsearching, the Checklist for the Development and Assessment of Cost-of-Illness Studies by Mueller et al. (Supplementary Table 2) (14). This scoping review revealed that most of the studies predominantly applied quality or critical appraisal tools that are intended for the assessment of full economic evaluations to assess the quality of COI studies. For example, eight studies identified in the scoping review reported to have used (part of) the BMJ Checklist, five studies used the CHEERS Checklist, four studies used the Drummond Methods, three studies used the Drummond 10-point Checklist, and another three studies used the CHEC-list. Some studies reported to have used more than one checklist or other tool/source; in part to develop their own checklist based on existing tools or guidelines. Where checklists and guidelines for full economic evaluations were applied, many studies only adopted a subset of the questions included in the checklists or guidelines for full economic evaluations.

Only two of the identified tools are designed for the assessment of COI studies: the Guide to Critical Evaluation by Larg and Moss, and the Development and Assessment of Cost-of-Illness Studies by Mueller et al. Where these tools were applied, studies mostly modified the original checklist by removing some questions or changing the wording of questions.

Some of the studies reported to have developed their own ad hoc checklist for their study or systematic reviews (which simply had a one-off purpose) based on existing guidelines, previous studies, and/or health economic guidelines.

### Assessment and Comparison of the Different Checklists and Their Questions

The matrix analysis charting all the questions and subquestions included in the identified checklists showed similarities in terms of key areas (dimensions) covered in the checklists such as study characteristics, the detailed methods that were used in the cost analysis, and how the study had been reported. There were some key differences between the checklists for full economic evaluations and the two tools specifically designed for COI studies. The latter two are more extensive and require the user/researcher to look at COI studies in more (technical) detail. The number of questions (criteria) in each checklist was recorded: the BMJ Checklist (*n* = 35), the CHEC-list (*n* = 19), the CHEERS checklist (*n* = 24, or *n* = 27 when including subquestions), the Drummond 10-point checklist (*n* = 10), and the Drummond Methods for the Economic Evaluation of Health Care Programmes (*n* = NA), the Guide to Critical Evaluation by Larg and Moss (*n* = 37), and the Checklist for the Development and Assessment of Cost-of-Illness Studies by Mueller et al. (*n* = 35) (Supplementary Table 2).

### Development of a Checklist for COI Studies

Following the assessment of the questions and subquestions in Step 2, a list of key questions relevant and applicable to COI studies that would need to be included in a checklist was developed, and the CHEC-list was used as a foundation for further development. The CHEC-list was chosen as a foundation because a rigorous process had been followed to build the checklist. This process included literature searches, taking into consideration existing health economic checklists and criteria, and building consensus using Delphi methods involving a panel of international experts. In addition, the CHEC-list is concise and comprehensive in its format as well as manageable with a total of nineteen questions. This was considered an advantage as the aim of this study was to develop a checklist for COI studies that is concise but comprehensive and can be expanded, where needed. Due to the fact that the CHEC-list is intended for full economic evaluations, the author team reviewed all nineteen questions in terms of their applicability and relevance to COI studies. We excluded those questions that were only relevant for full economic evaluations (e.g., a description of competing alternatives; an identification, measurement, and valuation of relevant outcomes for each alternative; information on an incremental analysis of costs and outcomes). This resulted in a preliminary list of fourteen questions applicable to COI studies (Supplementary Table 3). Findings from a previous study comparing the original CHEC-list to two other checklists (the BMJ checklist and the Quality of Health Economic Studies (QHES) checklist) suggested that the original CHEC-list is missing a question assessing whether study limitations are specified (24). Hence, the author team added a question on study limitations to the preliminary checklist for COI studies, resulting in a total of fifteen questions for the preliminary checklist. The order and wording of the fourteen questions were kept almost identical to the original CHEC-list as health economists and other experts working with COI studies that were to be interviewed were likely to be familiar with the questions and terminology, and this was considered helpful for the interviews. The questions could (preliminarily) be divided into the following three dimensions: study characteristics; methodology and cost analysis; and results and reporting.

### Expert Interviews

Between October 2020 and April 2021, 21 professionals (eleven male, ten female) from eleven different countries and with expertise in health economics (*n* = 17), economics, (*n* = 1), health policy (*n* = 2), and psychology (*n* = 1) participated in the interviews and provided feedback on the checklist. Experts were affiliated with academia, international policy organizations, governmental organizations, and consulting firms. More detailed information on the interview sample is provided in a separate paper https://doi.org/10.1017/S0266462323000181.

This study reached data saturation and consensus after those 21 interviews, finding similarities across those interview findings with little to no new findings emerging. Overall, experts were in favor of the checklist and expressed the urgent need for a checklist for COI studies. They suggested to remove, add, or rephrase some of the questions. Their feedback was considered and discussed carefully to finalize the checklist (before further presenting this checklist to experts at international health economic conferences). A more detailed analysis of the interview findings including relevant quotations is provided in a separate paper https://doi.org/10.1017/S0266462323000181.

### Finalization of the Checklist

Following expert feedback and discussions with experts at international health economic conferences, the final version of the checklist was agreed upon. The final checklist comprised 17 main questions (and some additional subquestions) across three domains: study characteristics; methodology and cost analysis; and results and reporting (Table 1). These domains are briefly described below.

*Domain 1 – Study characteristics:* This dimension aims to assist the user of this checklist in assessing whether a COI study formulated an objective (Item 1.), described the characteristics of the study population (Item 2.), and is explicit about the perspective chosen for the cost analysis (Item 3.).

*Domain 2 – Methodology and cost analysis:* This dimension aims to assist the user of this checklist in assessing whether a COI study reported their choice for their epidemiological approach (Item 4.), costing approach (Item 5.), and data collection approach (Item 6.) as well as whether it stated which resources their study identified (Item 7.), measured (Item 8.), and valued (Item 9.). It also guides the user in assessing whether a COI study stated their time horizon for analysis (Item 10.), reported whether they discounted future costs (Item 11.) and conducted sensitivity analysis/analyses (Item 12.).

*Domain 3 – Results and reporting:* This dimension aims to assist the user of this checklist in assessing whether a COI study presented their study results by cost category/sector (depending on their study perspective) (Item 13.) and discussed the generalizability of study results (Item 14.), study limitations (Item 15.), and ethical and/or distributional issues(Item 16.). It also asks whether the study reported any conflict of interest (Item 17.).

### Application of the Checklist

Based on the consensus built throughout the interviews and at international conferences, the following answer categories are suggested to be applied when answering the questions in the checklist: *Yes, Partially, No, Not Applicable (NA)*, or *Unclear.* The checklist contains one column listing all questions and subquestions, one column to note down the answer, and one column to add *Supporting Information.* Users are advised to extract relevant information from COI studies when answering the questions to support or justify their answer narratively and to increase accountability. Further detail on the above answer categories is given in the guidance (Table 2), and the reasons for choosing intermediate categories are summarized and published in a separate paper https://doi.org/10.1017/S0266462323000181.

It is considered sufficient if one reviewer completes the checklist, assuming that they use the data extraction column to add information that justifies their answer. It is recommended to seek out to a second reviewer where information is not clear, and discrepancies need to be discussed. This checklist does not require the user to add scores to their answers or produce a total score for each study and a ranking of studies by score. Where desired or needed, it is however open to and possible for users to add scores to their answers (e.g., *yes* = 1, *partially* = 0.5, *no* = 0). When answering the questions as suggested in the guidelines, users will be able to identify the number of *yeses* or *nos*, which could give them an idea of the comprehensiveness of the study and an opportunity to reflect upon the reasons for a certain indication.

Where needed, the checklist can be modified and/or expanded, but it is suggested to clearly report any modifications or expansions to maintain consistent use.

### Development of Guidance Statements

Additional guidance was developed describing the purpose and meaning behind each question and listing examples of best practice, see Table 2.

## Discussion

The aim of this study was to develop a consensus-based checklist that can be used as a minimum standard to appraise the comprehensiveness, transparency, and consistency of COI studies. This study is the first to establish a checklist in English to review and assess the methodological approaches taken and reported in COI studies. The checklist was developed with the engagement of international experts from relevant backgrounds such as health economics, health policy, and psychology who had conducted or provided guidance for COI studies. The checklist is based on existing checklists and guidelines for health economic studies, expert qualitative interviews, and feedback from stakeholders at international health economic conferences. It is a pragmatic, generic, concise, and comprehensive tool that can be applied in several scenarios and can be considered a minimum standard, for instance, when reviewing and assessing COI studies for example as part of a systematic review or when building an economic model.

Additionally, this study addresses the inconsistency in the use of checklists and guidelines to appraise COI studies and provides further evidence that there is an urgent need for a standardized checklist to review and assess COI studies. It is the first study to fill this gap and provide a tool that could be used by users/researchers more consistently and internationally.

### Comparison to Other Checklists

The checklists identified in the scoping review of this study cover similar questions and show methodological parallels. Those checklists, in particular the CHEC-list, provided a starting point to the development of a consensus-based checklist for COI studies available in English. The Guide to Critical Evaluation by Larg and Moss and the Checklist for the Development and Assessment of Cost-of-Illness Studies by Mueller et al. are designed for COI studies but may require a deeper level of technical and methodological detail and are extensive in length. It was unclear as to how the guide by Larg and Moss had been developed and whether it is based on expert opinion and consensus (25). The checklist by Mueller et al. was developed using expert opinion but it was first and foremost established for the German context and is officially only published in German (14). Both tools have been taken into consideration for the development of the present consensus-based checklist for COI studies.

### Strengths and Weaknesses of the Study

This study established and followed an extensive, structured, and iterative approach to the development of the checklist, involving literature searches, expert interviews, and further discussions among experts at international health economic conferences. The involvement of twenty-one international experts working in health economics, health policy, and psychology, and users as well as developers of existing quality or critical appraisal tools is one of the key strengths of this study. The checklist is a generic tool that can be applied to different disease areas and scenarios. A potential weakness of this study is that the checklist has not yet been formally pilot-tested. The checklist has, however, been applied by staff and students at Maastricht University and the University of Birmingham who have provided constructive feedback. The author team will initiate further piloting and testing of the checklist across different disease areas, and potentially refine its criteria, where relevant. This will be undertaken by students and researchers (initially by the authors and their research groups) using this tool for reviewing and assessing COI studies as part of future systematic reviews. It is planned that the checklist will be published on a university Web site (Maastricht University) alongside other quality and critical appraisal tools. This will allow us to monitor use of the checklist and to provide details on correspondence, in order to collect feedback from a wider range of users (outside of our research groups). Another limitation of this study is that due to the iterative process of development, the feedback collected during the interviews and the feedback collected at conferences were merged for analysis, making it difficult to compare the changes made based on the interviews and those made due to conference discussions. Further, the use of the CHEC-list as a starting point may be a limitation due to the CHEC-list being developed for full economic evaluations. However, other tools and checklists including the guide by Larg and Moss and the checklist by Mueller et al. were carefully considered during the development of the new checklist.

## Conclusion

There is currently no standard checklist for the critical appraisal of COI studies and, as a result, the use of checklists for COI studies is inconsistent and heterogeneous. The consensus-based checklist for COI studies is a first step toward standardizing the critical appraisal of COI studies and is one that could be considered a minimum standard. The consensus-based checklist can help to improve comprehensiveness, transparency, and transparency in COI studies, to address heterogeneity and to enable better comparability of methodological approaches across international studies.

## Supplementary material

For supplementary material accompanying this paper visit https://doi.org/10.1017/S0266462323000193. click here to view supplementary material
