# Supplementary material for: A consensus-based checklist for the critical appraisal of cost-of-illness (COI) studies
Source: Int J Technol Assess Health Care. 2023 Jun 16;39(1):e34. doi: 10.1017/S0266462323000193 (PMC11574538; doi:10.1017/S0266462323000193)
Supplement: Supplementary file 1 [file S0266462323000193sup001.docx]

Supplementary Table 1

Search strategy for Medline (Ovid)

| cost-of-illness.mp. or "Cost of Illness"/  burden-of-illness.mp. or "Cost of Illness"/  (cost of illness).m_titl.  cost.ti,ab.  costs.ti,ab.  (economic burden).ti,ab.  (burden of disease).ti,ab.  AND  Systematic Review.mp. or "Systematic Review"/  Review.ti,ab.  checklist.mp. or Checklist/  quality assessment.mp. |
| --- |
